# Beyond the lab: a nanoimprint metalens array-based augmented reality

**DOI:** 10.1038/s41377-024-01429-x

**Published:** 2024-05-06

**Authors:** Chi Li, Haoran Ren

**Affiliations:** School of Physics and Astronomy, Monash University, Melbourne, VIC Australia

**Keywords:** Imaging and sensing, Integrated optics

## Abstract

A see-through augmented reality prototype has been developed based on an ultrathin nanoimprint metalens array, opening up a full-colour, video-rate, and low-cost 3D near-eye display.

Integral imaging (II) display, a form of light field display utilising a lens/pinhole array to capture and reproduce the light field, was invented by a Nobel laureate Gabriel Lippmann more than a century ago^1^ This technique reconstructs the entire image through a large array of small lenses, resembling the mechanism of a fly’s eyes. The resulting images encapsulate the full light field information of the original three-dimensional (3D) object, akin to holography. Unlike holography, this technology is not constrained to coherent light sources. II display inherently offers properties such as full parallax and quasi-continuous viewpoint, presenting a genuine 3D display that circumvents visual fatigue, a common issue in binocular parallax-based 3D displays due to the vergence–accommodation conflict^2^.

The progression of II display was slow before the twenty-first century due to technological limitations, but has rapidly advanced, especially in the past decade, owing to enhanced algorithms, improved fabrication capabilities and high-speed digital cameras. Flat meta-optics represents a promising candidate for the next-generation 3D display technology, with ultrathin metalenses emerging as an ideal alternative to conventional bulky lenses^3–6^. Metalenses exhibit an unprecedented capacity to manipulate light at a subwavelength scale, encompassing the precise control over the amplitude, phase, polarisation, and dispersion of transmitted or reflected light from dielectric or plasmonic meta-atoms^7,8^. Recently, metalenses have shown great potential for II display, addressing critical chromatic aberration encountered by traditional microlens arrays. However, fabricating a large-scale metalens array and its integration with a commercial micro-display for II display have remained a challenging task. Besides, computational algorithms used for encoding 3D objects and creating elemental image arrays have remained too slow to achieve real-time rendering of 3D objects for practical video-rate II displays.

In a recently published paper in *eLight*, a research team led by Professor Zong Qin and Professor Jian-Wen Dong from Sun Yat-sen University has introduced the use of a large-scale nanoimprint metalens array for 3D near-eye II displays^9^. This system combines a large-scale metalens array, a commercial micro-display, and a real-time rendering algorithm, capable of producing high-quality 3D images with motion parallax and focus cues (Fig. 1). Their full-colour, real-time, see-through meta-prototype highlights the use of their device for practical virtual and augmented reality (VR and AR, respectively). The researchers employed the nanoimprint fabrication technology^10^ to fabricate a large-scale (1.84 mm by 1.84 mm) metalens array in an adhesive material with a refractive index of 1.9. A 4-by-4 high-quality metalens array was integrated with a commercial micro-display via a 3D-printed holder. To achieve video-rate II displays, they also introduced a new rendering method exploiting static mapping between voxels and pixels in the II display. This rendering method allows real-time performance through a lookup table, bypassing traditional geometric projections. Verification of the meta-system’s true-3D display capability, they also demonstrated a see-through prototype capable of integrating 3D images with surrounding objects, demonstrating the AR application.

**Fig. 1:**
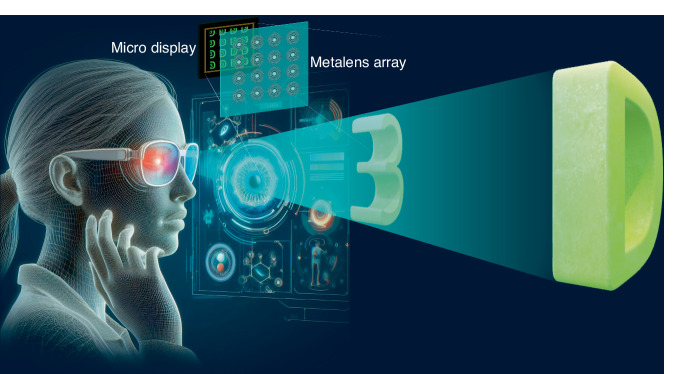
A conceptual illustration of a nanoimprint metalens array-based AR

Even though nanoimprint lithography for high-throughput metasurface fabrication and real-time rendering algorithm could propel the development of II displays for future VR and AR applications, several challenges persist in this field. For instance, high-resolution image acquisition remains a huge hurdle, demanding micro-display sensors with ultrasmall pixel sizes down to sub-micrometre scale. However, manufacturing such small sensors poses a considerable challenge. In this context, time-multiplexed light fields with high refresh rate monitors may offer a viable solution^11^. Secondly, the refractive index of available nanoimprint adhesives remains low, necessitating high-aspect-ratio nanopillars to build up metalenses, incurring the shadowing effect that could reduce the diffraction efficiency of high spatial frequencies. Thirdly, development of truly interactive 3D displays demands the use of dynamic metasurfaces for fast tunability and low power consumption. Although various mechanisms such as phase change^12^ and the electro-optic effect^13^ have been proposed to realise dynamic metasurfaces, this development is still in its infancy. Notably, the unique ability of metasurfaces to interact with multiple degrees of freedom of light, such as polarisation^14^, wavelength^15^, orbital angular momentum^16^ and spatiotemporal beams^17^, could open the door to further enhance the dynamic function and image capacity of metasurface-based displays. Apart from the hardware efforts, the rapid development of machine learning, neural networks and artificial intelligence could facilitate the software aspect of future 3D display technology.
